# Governing beyond the project: Refocusing innovation governance in emerging science and technology funding

**DOI:** 10.1177/03063127231205043

**Published:** 2023-11-16

**Authors:** Robert DJ Smith, Stefan Schäfer, Michael J Bernstein

**Affiliations:** 1 University of Edinburgh, Edinburgh, Scotland, UK; 2 Research Institute for Sustainability–Helmholtz Centre Potsdam, Potsdam, Germany; 3 Austrian Institute of Technology GmbH, Vienna, Austria; 4 Arizona State University, Tempe, USA

**Keywords:** innovation governance, responsible innovation, research funders, institutions, experimental collaboration, the project form

## Abstract

This article analyses how a recent idiom of innovation governance, ‘responsible innovation’, is enacted in practice, how this shapes innovation processes, and what aspects of innovation are left untouched. Within this idiom, funders typically focus on one point in an innovation system: researchers in projects. However, the more transformational aspirations of responsible innovation are circumscribed by this context. Adopting a mode of critique that assembles, this article considers some alternative approaches to governing the shared trajectories of science, technology, and society. Using the idea of institutional invention to focus innovation governance on four inflection points—agendas, calls, spaces, evaluation—would allow funding organizations and researchers to look ‘beyond the project’, developing new methods to unpack and reflect on assumed purposes of science, technology, and innovation, and to potentially reconfigure the institutions that condition scientific practice.

## Innovation governance and its discontents

In the 21st century, the demand for policy instruments that ‘bring science and society into a common frame’ has grown (Rabinow & Bennett, 2012, p. 134). Talk has proliferated of such things as mission-oriented innovation policy, co-creation, inclusive innovation, transformative innovation policy, grand challenges, and responsible innovation. The official documentation announcing Horizon Europe, the ninth instantiation of the European Commission’s seven-year research and innovation framework programme, is illustratively anodyne. Announced in 2018, the 95-billion-Euro scheme claims to adopt a ‘mission-orientation’, ‘co-designed with citizens, stakeholders, the European Parliament and Member States’, to ‘tackle issues that affect our daily lives’ such as ‘the fight against cancer’, ‘clean transport’ and ‘plastic-free oceans’ (European Commission, 2018, pp. 3–6). Contemporary public investments in science, it would seem, should be made strategically and inclusively to address the most pressing grand challenges facing the world today.

These developments show how science and innovation are changing as objects of governance. No longer viewed as self-directing, they are seen—with varying degrees of clarity—as things to be chaperoned by state and non-state actors (Schot & Steinmueller, 2018). This collectively held assumption is encapsulated in the phrase ‘innovation governance’, the idea that the shared trajectories of science and society are malleable, uncertain, and convergent, and can be scoped, interrogated, and actively debated by broad groups of people. Innovation governance shifts the object of scrutiny away from risk to ‘upstream’ concerns about the framing, purpose, and appropriate ‘mixes’ of different kinds of science and innovation. It has circulated at the interface of science and technology studies (STS) and science policy, in prominent documents such as the expert report *Taking European Knowledge Society Seriously* (Felt et al., 2007) and has been carried into international think-tanks such as the Organization for Economic Cooperation and Development (Frahm et al., 2022).

The turn to innovation governance has placed public funding organizations in the spotlight and many have been key experimental spaces in the development and institutionalization of the idea. Also in the spotlight are researchers in the critical social sciences who have been central to developing ideas and practices within the frame of innovation governance, particularly when emerging technologies are invested in and publicly contested. However, many subjects of such policies—often the same researchers who have argued for them—have expressed unease. Several have suggested that the language of innovation governance mobilizes disparate fields of social scientific study and legitimates interventions, without changing who gets to make decisions about innovation or requiring incumbent actors to go about doing anything new (Ribeiro et al., 2017). Others question whether funders’ approaches adequately mesh with the realities of academic life and established meanings of scientific responsibility (Davies & Horst, 2015; Delgado & Åm, 2018; Felt, 2017). Rather than being ‘cared for’, these ideas become things to be ‘taken care of’ under the auspices of ethics, public acceptance or impact (Evans & Frow, 2015), often by junior social scientists attached to ambitious technoscientific projects (Lyle, 2017; Viseu, 2015). The ‘rhetoric’ is criticized for failing to challenge established and widespread ideas about the relationship between science and society, which render the broader frames of innovation off-limits (Hartley et al., 2017). Instead, activities claiming to further a ‘responsible and inclusive science’ replace political and citizen engagement, while simultaneously making it harder to challenge the scientific endeavour (Ledingham & Hartley, 2021).

While valuable, these accounts are fragmented and often situated in terms of localized experiences (Hilgartner et al., 2016). As others have begun to argue, there is a need to thread these disparate works together and examine how the dynamics they highlight emerge from particular arrangements of people, organizations and ways of thinking (Åm, 2019; Felt, 2017; Joly, 2015). But we also need to go further, to see ‘new’ forms of innovation governance—and the critical social scientists enacting them—as implicated in the co-production of particular kinds of science and politics, embedded in longer trajectories of thought about how to govern science, technology and innovation in the public interest (Frahm et al., 2022; Irwin, 2006).

Here we analyse similarities in how innovation governance takes shape in different settings and unpack what it is that produces them. Our goal is to adopt a mode of critique that assembles—that might offer ways in which science administrators, scientists, and social scientists could reconfigure the landscape of science and policy to produce different kinds of governance (Calvert & Schyfter, 2017; Latour, 2004). We therefore ask two questions of the situation, one interpretive and one normative. First, what institutional configurations and policy instruments prevail as new idioms of innovation governance are mapped onto entrenched practices of public policy creation? And second, what institutional changes, if any, might chaperone the shared trajectories of science, technology, and societies in more equitable, environmentally sensitive, and democratic ways than currently prevail?

We focus on how responsible innovation has been used by European, U.S. and British research funding organizations to govern emerging science and technologies.^1^ Drawing on insights, methods, practices and approaches developed in and around STS (Ribeiro et al., 2017), responsible innovation has been most clearly articulated as an attempt to ensure that science and policy actors enact a ‘collective care for the future’ (Stilgoe et al., 2013). This is considered achievable by: (i) scoping the potential intended, unintended and even unknowable changes to social and political order that might emerge as a result of bringing new knowledge or technologies into the world; (ii) reflecting on the motivations driving investment in new science and technology; (iii) opening-up these discussions to relevant experts and citizens; and (iv) incorporating the resulting appraisal into governance processes (Macnaghten, 2020). This resonates strongly with definitions offered by, for instance, von Schomberg (2013), who was at the time working in the European Commission.

As we detail below, responsible innovation belongs to an idiom of innovation governance that has gained currency in Western research funding organizations in the past decade. We see the phrase as specific yet commensurate with a range of other terms that describe recognizably similar ideas in innovation governance—human practices, anticipatory governance, real time technology assessment, upstream engagement, or constructive technology assessment, for instance. While these ideas each have their idiosyncrasies, they exhibit clear family resemblances and can be distinguished from other idioms that have shaped past eras of innovation governance, such as ‘applied research’ or ‘mode-2’ (Flink & Kaldewey, 2018). We thus see the uptake of responsible innovation and its use in a range of different contexts as illustrative of a broader tendency, in response to a shared set of concerns (see Felt, 2017; Rabinow & Bennett, 2012) within the governance of science, technology and innovation, without being strictly representative in a formal sense.

We draw directly from a series of published accounts and semi-structured interviews with 47 people involved in the development and practice of responsible innovation. Six interviewees were natural scientists, 33 were humanities scholars or social scientists and nine were science administrators. Twenty-nine were based in the U.K., two were based in the U.S., and 16 were based in the E.U. All researchers were based in universities and research institutes—important sites of responsible innovation practice—and most worked on emerging technoscientific endeavours such as synthetic biology, new battery technologies or big data and machine learning. Interviews were guided by a schedule designed to elicit accounts of the lived experiences of responsible innovation both in specific projects and in policy settings. We asked about the organizational context, interviewees’ methods, their explicit or implicit notions of change, and whether any outcomes were visible.

These data were supplemented with participant observation in research funding organizations developing responsible innovation policies from 2018-21, and discussions in three workshops to explore key theories, methods, and sites of governance for responsible innovation. We traced the interpretation of this idea through funding organizations and into the sites of governance they demarcated as they gave meaning to it, where we then sought to understand the significance of funders’ decisions for other related actors. For instance, were new possibilities for collaborative research between the natural and social sciences being created? How did researchers interpret their mandate to ‘intervene’ in the development of science and technology? And what tensions emerged from differences in the meanings given to responsible innovation by various actors? Our collaborations with funding organizations centred on three funding programmes on nanomedicine, materials science, and biotechnology, each a multilateral scheme with public funders across Europe. We initially explored our empirical material inductively and collaboratively with participants in the three workshops. From these explorations, the use of responsible innovation to couple researchers to scientific projects emerged as a recurring observation and point of discussion across our empirical contexts. After noticing this, we conducted a thematic analysis of interview transcripts to examine the social dynamics in more detail.

Our analysis shows that one specific configuration of governance, centred on ‘the project’, has come to dominate at the expense of a broader set of possibilities. Philosophers have long argued that modern science is indexed through ‘the project form’, a construction that holds together long enough for verifications to be produced (Bachelard, 1984, p. 11). However, the projects constituting contemporary science are also bureaucratic entities through which various actors’ practices become governable. While there is a growing literature on the ‘projectification’ of science and its downsides, we mobilize the term symmetrically—as a concept that defines a certain set of identities, relationships, and points of passage in response to a specific problem definition. Our approach follows Vermeulen (2015) and Felt (2016), who characterize projects as temporary organizational entities, distinct from universities, research groups and funding agencies, that demarcate specific temporal and spatial boundaries in which work can occur and be evaluated. They are a means through which funders and researchers partition diffuse agendas into manageable chunks with discrete obligations. Projects function as templates, an easily replicable form of social organization that, in recent developments around making science and technology development more responsible, have in an act of delegation by funders been made to embody the appropriate and sometimes singular site for enacting responsibility.

Our central argument is that a reliance on projects as the primary site for innovation governance keeps the more transformative aspects of the idea at bay. If the proclaimed goals of contemporary innovation governance are to be taken seriously, there is a need to move ‘beyond the project’ and reformulate the targets of governance within research funding organizations, to shift attention away from the outputs of governing through projects—the things being done and made by these researchers—and toward the practices, habitual patterns of thought, and policy instruments that enable, shape and sustain them. Drawing from social studies of governance to introduce a concept of ‘institutional invention’, we specify four inflection points—agenda setting, funding calls, spaces, and evaluations—where research funding organizations in collaboration with researchers in the critical social sciences might foster more substantive instantiations of innovation governance.

## Research funding organizations and the governance of emerging technologies

Western funding organizations have sought to govern science, technology, and innovation in response to concerns about the relationship between science and society. To do so, they have used a specific idiom of innovation governance of which responsible innovation is a part. We trace what responsible innovation becomes as science administrators, policy makers and social scientists use experiments, policies, assessments, speeches, committees, and various other tools of governance. We pay particular attention to the ways in which administrators in organizations translated, filtered, and domesticated responsible innovation to align with existing modes of working, routine ways of thinking, and established forms of governing. Using illustrations from the work of different funding organizations, we describe a familiar cycle of contestation leading to brief periods of institutional experimentation and responsiveness, followed by longer periods of domestication in which the idea travels from one locality to the next.

Since their creation as part of the infrastructure of late-modern science at the turn of the nineteenth century, funding organizations have played central roles in governing science, technology, and innovation. Developing from models such as the Rockefeller Foundation and Carnegie funds, post-war organizations like the US National Science Foundation have long-operated as intricate bureaucracies for planning, strategic management, and ‘rational’ world building in the name of what states define as the public good (see Jacobs, 2019). They sit at the intersections of governmental agendas, scientific communities and political discourses, which administrators work to navigate (Fisher & Maricle, 2015; Kearnes & Wienroth, 2011; van der Burg, 2010). They define the bounds of emerging fields, shape academic trajectories, provide science advice to government and contribute to public discussion of science and technology (Kearnes, 2013; Rabinow and Bennett, 2012; Wehrens et al., 2022; Weisz et al., 2017). And as vernaculars and methods of ‘evaluation’ and ‘audit’ spread far beyond their origins in modern finance, science funders, too, developed modes of governing that construct the scientific self as an entity that can be assessed in the same categories used to assess financial performance (Strathern, 2000). Funders thus define the criteria of academic achievement and identify the individual as its agent.

While they usually work quietly and out of public sight, various controversies and disasters over the course of the twentieth century have sensitized most funders to the social, ethical, and political dimensions of their work, particularly as they have taken on responsibility for managing the relationships between science and its various publics. In this context, developments in the life sciences and biotechnology have come under sustained scrutiny: Recombinant DNA experimentation, cloning and genetic modification, DNA sequencing, and stem cell research are examples. More recently, however, the purview has expanded to include almost any new promissory technoscientific endeavour set to receive large public investments, including genomics, nanoscience, brain science, synthetic biology, geoengineering, and most recently artificial intelligence. These fields exist within a particularly acute political economy of ‘techno-economic promises’ (Joly et al., 2010) but are also argued to be inherently uncertain with regard to their potential impacts (Stilgoe et al., 2013). Their initial investments are often acute moments of public contestation, as civil society groups and scientists alike question both their potential impacts and epistemic credibility (Mahfoud, 2021). Further, because they are new, sociologists of technology have argued that their trajectories are particularly up for grabs and definable through public debate because lock-in has not yet occurred. These fields of emerging science and technologies have been key sites for the development of innovation governance in the last two decades.

Controversies often open up assumptions and value judgements for scrutiny (Rip, 1986) but they also create ‘institutional voids’, situations in which there is no clear polity or process for producing it (Hajer, 2003). In these moments, funders and other agents of government (see Rothstein, 2013) have had to experiment and develop new repertoires as they learn how to manage the ethical, social, or political dimensions of the fields in which they are investing, while also achieving their operational objectives. It was out of cycles of promise, contestation, and investment that in the mid-2000s responsible innovation emerged in communities of STS scholars studying the development of emerging technologies such as nanoscience, synthetic biology and geoengineering (Guston, 2007; Ribeiro et al., 2017). Moments of reflection and experimentation sparked by institutional uncertainty drew critical social scientists into spaces of policy, and made the ideas behind responsible innovation salient to policy makers and administrators in the U.S., the U.K., and continental Europe, among other places (see Fisher, 2019; Marris & Calvert, 2020; Owen & Goldberg, 2010; Rip, 2016; Wilsdon et al., 2005). The phrase circulated amongst a range of other commensurate terms such as anticipatory governance, constructive technology assessment, real time technology assessment, responsible development of technology and upstream public engagement before being codified in a variety of policy frameworks by research funding organizations, often in collaboration with these same scholars (Doezema et al, 2019; Macnaghten, 2020; Owen & Pansera, 2019).

However, over time, earlier moments of reflection and invention closed down as administrators replaced the practices they piloted with established organizational repertoires and incumbent modes of governing. In Europe, for instance, Rip (2016) recounts how von Schomberg’s vision for RRI struggled to gain purchase amongst a range of competitors that included ‘citizen science’, ‘six keys for responsible innovation’ and ‘the three Os’—open innovation, open science and open to the world. By the mid 2010s, the European Commission de-prioritized deliberative public events occurring prior to research and studies of its own institutions, and instead began to emphasize citizen participation within the sites of knowledge production (Macq et al., 2020; Rayner, 2012). Citizens were positioned as ‘citizen scientists’, as active producers of science and technology rather than decision-makers about how science and technology should be governed (European Commission, 2017a). In the 2018-2020 Horizon 2020 work plans, RRI was discussed as a cross-cutting theme, but individual programmes were left to operationalize the idea of responsible innovation in their own terms, with little guidance and varied outcomes (Novitzky et al., 2020). Although some European programme administrators recognized that responsible innovation demanded ‘something different’ from what had come before (e.g. Smith et al., 2021), it has commonly been used as a direct replacement of Ethical, Legal, and Social Implications (ELSI) initiatives, with administrators simply relabelling the respective sections of their application forms. In the U.S., subsequent investments in high technologies have largely departed from the methodologies developed around the National Nanoscience Initiative (Fisher, 2019). And in the U.K., despite these internal governance experiments and the rhetoric of ‘collective responsibility’, almost all responsible innovation work in fields such as synthetic biology, nanoscience and data science has been delegated to research projects rather than adopted at operational funding levels and strategic decision-making processes. This has happened against a background of research council staff struggling to situate responsible innovation in their administrative practices and in relation to dominant political logics of economic growth, academic independence, and a need to accelerate innovation (Owen et al., 2021; Smith et al., 2021).

What remains of this process of domestication is an instantiation of responsible innovation that has been integrated into a project-driven mode of governing—a mode that has long been central to research and innovation policy but which has expanded in the past twenty years (see, e.g. Felt, 2017; Gläser & Laudel, 2016; Hall, 2019; Lepori et al., 2007). This is evident in that the dominant instrument used by research funders to enact the ideas behind responsible innovation has been the simultaneous funding of humanities and social science research with the natural sciences and engineering through competitively allocated projects. Most famously operationalized as the ‘Ethical, Legal and Social Implications’ programme of the Human Genome Project, one now finds translations of this institutional configuration in programmes around the world, including the US NSF and NIH, Genome Canada, the European Commission, Australia’s Commonwealth Scientific and Innovation Research Organization, the Norwegian Research Council and several of the UK Research Councils (Hilgartner et al., 2016). However, whereas early programmes typically operated as distinct funding streams and explicitly at arms-length to natural scientific agendas, current instantiations under the label of responsible innovation often co-fund natural and social science in shared projects, charging the social sciences with a mandate to ‘intervene’ in the trajectory of the natural sciences (Rodríguez et al., 2013). An idiomatic example is the NSF’s ‘Understanding the Rules of Life’ programme on synthetic cells, which mandates that each application include ‘at least one bioethics researcher’ (NSF, 2018, p. 4).^2^ This vague requirement also points to the ambiguity in terms of outcomes that such funders want to achieve through integration.

Central to these dominant enactments of responsible innovation is the idea that the most appropriate people to address the questions of innovation governance are researchers, the most appropriate sites for concerns to be addressed are laboratories, centres and universities, and the most appropriate way for this to be achieved is by mandating that researchers demonstrate they take such questions seriously by building responsible innovation ‘components’ into their competitively awarded research projects. In the next section we present a thematic analysis of concerns with the ways in which responsible innovation has been operationalized by research funding organizations in this project-driven mode. Although the local settings are obviously diverse, our claim is that the institutional configuration of contemporary science policy is contiguous enough that a series of core issues emerge.

## Project level collaboration and institutional excess

Having situated the operationalization of responsible innovation within the policy landscape, we can begin to weave published accounts with vignettes from our qualitative interviews to explore the broader processes through which science and technology projects are given their contemporary shape. We make explicit how the identities, relationships, and points of passage that characterize projects today are established in a manner that keeps at bay the more transformative ambitions of responsible innovation. Our goal is to enable a critique not just of individual projects, but of the culture that produces them by highlighting three processes: framing, valuing, and partitioning.

### Framing

Frames develop in the interactions of social groups (Eden, 2004; Pinch & Bijker, 1984). By bounding certain dimensions of a situation, they provide ways of conceptualizing problems and locating solutions. To take a simple but relevant example, if food security is framed as a problem of low crop yields, technologies to increase those yields, such as genome editing, can be presented as a solution. If, however, food security is framed in terms of farmers’ access to and control of their production methods, then modifying land ownership and seed licensing agreements become more plausible solutions (Helliwell et al., 2017). We can quickly see how one frame may lead actors to prioritize technological solutions while another frame may orient people towards political or social solutions. Frames are not inherently incommensurable but there will always be multiple ways of framing problems and solutions.

Several interviewees explained how their projects were constrained by the frames established by research funders. One researcher working on an energy project in low resource settings noted:

Something I find frustrating … is that when we did engage with communities actually their biggest priority was water. It wasn't energy, but ultimately we were funded to deliver on energy. So we were having to say to them, ‘Okay that’s great. And we’ll do our best to connect you up with some water charities. And let’s talk about how energy might help you with your water problems’, but ultimately in an energy project. … So you apply to do a specific thing and that was the case with this particular call. (Lecturer, STS)

Here, the frame established by the funding programme limited the capacity of the project to respond to community priorities. The researcher points to ways in which they were able to manoeuvre within the confines of the institutional configuration, by locating community priorities for water availability within the project’s frame of energy supply. But as another participant noted, this ‘pre-framing’ of the project limits the capacity to open up discussion about goals of technology:

Responsible innovation is often framed in terms of individual technologies, when in fact, what the [social] scientists have been calling for is actually opening up understandings of the problems to which these products are meant to be solutions. (Senior lecturer, STS)

Many interviewees were deeply sensitized to the wider political frame that surrounded their projects. This was raised in the context of data science, genomics, neuroscience, and robotics but was clearest for synthetic biology in the U.K. To constitute the field in the late 2000s, a relatively small group of scientists and policy makers sought to build a frame in which the worth of synthetic biology was its capacity to drive technological advances that would become marketable products to address a range of social and environmental ills (Hilgartner, 2015; Marris & Calvert, 2020). When David Willetts, then UK Minister for Science, stood on stage at an international synthetic biology conference in London to announce his government’s £60m investment through a ‘Synthetic Biology for Growth’ programme, he announced: ‘Synthetic biology has huge potential. Indeed, it has been said that it will heal us, feed us and fuel us’ (Willetts, 2013). During these years, and the years that followed, research funding organizations convened a series of semi-public spaces in which the vision of a future ‘bioeconomy’ was articulated, framing synthetic biology as a contributor to national economic competitiveness (Kearnes, 2013).

Analyses of funding programmes note that societal problems are most frequently framed in terms that foreground specific, often technical, solutions (Brooks et al., 2009), or are commonly developed within narrow socio-economic models of innovation (Joly et al., 2010). One senior researcher distilled the political economic challenge down to a ‘minimal condition of asymmetry’ for any social research attached to large scale technoscientific projects:

[W]e’re all being paid to be in this room to deliver a technology. We can deliver it more or less responsibly. We can deliver with more or less attention to social impacts or whatever jargon we want to use … but at the end of the day, what we’re being audited on is whether or not we make the technology and whether or not [it] is going to be taken seriously by the other social actors who might get in the way of the technology. (Associate professor, Anthropology)

Framing is fundamental to making projects doable. A principal investigator noted:

In terms of planning research, you can't start a project with a problem and then decide after a year or two that ‘oh we don’t need the automation people, actually we need political scientists.’ (Principal researcher, Ethics)

But the salient point is that within the dominant institutional configuration, health, food security and energy supply become problems amenable to commodity-driven solutions from laboratories, while social scientists are called upon to integrate sociality into these commodities or smooth the path to market. Responsible innovation thus ends up ‘working with the grain … meaning that difficult questions stop getting asked’ (Professor, Innovation Policy). Alternative forms of social scientific inquiry that question this frame are either entirely bounded out or must be laboriously re-articulated (Morris et al., 2019; Strathern & Khlinovskaya Rockhill, 2013).

### Valuing

Embedded within a frame are specific registers of worth. These registers are often implicit but are observable in the instruments used to measure, accelerate, assess, and partition academic practices (Felt, 2016). Beyond the epistemic case put forward for a project, grants are commonly evaluated on their plans for impact, science communication and outreach, data management, and ethical compliance. The careers of academics are evaluated in terms of impact factors and H-indexes, grant income and teaching feedback. A cumulative effect of such organizational reforms is that the ‘epistemic living space’—the space in which science, technology and innovation are produced—is increasingly crowded with competing demands (Davies and Horst, 2015; Felt, 2016; Fochler et al., 2016). One professor was blunt:

It’s very clear that there are massive institutional barriers to this way of working. Those are evaluation metrics and progression criteria. (Professor, Innovation Policy)

However, these evaluative regimes do not present themselves uniformly throughout a project’s lifespan. Several participants described how their roles were initially ambiguous. Despite funders seeing the idea of responsible innovation as appealing, there was no real sense of what it might mean in practice. Describing her early engagements with natural scientists, one social scientist noted that:

They didn’t know what they wanted, and they also didn’t necessarily want to put a massive amount of time into it, so that gave some freedom. That didn’t lead to tensions. Actually, … it was slow and there were no deliverables, no expectations. It was really low pressure, kind, friendly. We did poetry and stuff …. (Assistant Professor, STS)

In such situations, the social sciences are valued equivocally, but this equivocation can be productive (Fitzgerald et al., 2014), allowing researchers to ‘take a very gentle, and subtle approach’, aiming to ‘figure this out together’ and sometimes discovering ‘that there was actually quite a strong will from scientists to engage’ with this approach (Senior Researcher, Anthropology).

Nevertheless, interviewees often went on to narrate a narrowing of their investigative space as projects advanced, with bottom-up, collaborative approaches eventually being crowded-out. The social scientists in one research centre explained that their natural scientific colleagues generally understood the need for ‘the field of synthetic biology to pay attention to the issues raised by responsible research and innovation’ (Professor, Innovation Policy) and that their initial approach was ‘to keep it open and try not to define responsible research innovation or to perform a very specific set of practices scientists should engage in to demonstrate responsibility’ (Senior Lecturer, STS). But over time ‘generally, most people lost interest’ because ‘there are so many other pressures on them’ (Researcher, STS). These findings mirror those of Fochler et al. (2016), who found that the repertoires researchers use to talk about the goals of their research narrowed significantly in the period between PhD and post-doctoral work as they became imbricated within contemporary fabric of academic life, but they also suggest that such repertoires may narrow over the course of an individual project.

There are multiple points within a funding programme in which registers of worth are created, including the design of funding calls, proposal reviews and impact assessment. However, two instruments in which this is particularly crystalline are the annual and mid-term reviews of projects; obligatory passage points through which worth is defined and worthiness assessed. Perhaps the most infamous mid-term review is the National Science Foundation’s Engineering Research Committee’s Site Visit Team’s assessment of SynBerc’s Human Practices Thrust as being a ‘risk’ to the future of the centre’s success, in part because it did not present a coherent analytical picture and, in particular, did not adequately contribute to the biosecurity policy frame that had developed in the U.S. around synthetic biology following the 9/11 terrorist attacks (Rabinow & Bennett, 2012, p. 134). Several participants recounted less extreme but similar experiences. One junior scientist recounted being ‘constantly asked’ about responsible innovation, suggesting that it was in evaluative moments such as mid-term review that a particular vision of worthwhile practices began to emerge, and meaning that they had to tailor their contextualized enactments to mesh with a dominant one:

I think there’s been a little bit of a challenge in terms of trying to demonstrate that what we’re doing can fit within the more formal responsible innovation definitions. We think it can but we’ve just had to think quite carefully about how to word it. Particularly in relation to the research councils’ vision of it. (Researcher, Synthetic Biology)

The point here is not that interdisciplinarity is evaluated but that the register of worth used to evaluate it is one which tracks dominant political imaginaries of what science is for and how responsible innovation should be practised, in the process simultaneously disavowing alternative registers of worth and academic contributions, such as social analyses of science. As noted by Müller and de Rijcke (2017), an increasingly competitive science with quantitative indicators as the measures of success poses significant challenges to the espoused values of sustainability and responsibility in science, technology and innovation ostensibly geared towards missions, transformations, societal challenges and the like.

### Partitioning

Our final theme is partitioning. Organizing work into projects allows funders and researchers to partition diffuse research agendas into manageable chunks, allocating ownership to parts of extended scientific workflows and allocating valuable outputs, such as first-author articles, to individuals, such as PhD students (Hammang & Frow, 2019). Projects establish working relationships between partners who are equally time-limited and tied to the production of specific outputs. For instance, recounting her failure to create moments for reflection and debate amongst her scientific colleagues, this professor emphasized her obligation to her student:

I’m really impressed with [what my student is doing]. I’m very disappointed about the way [the scientists have] taken it up. … I know that if I had made an effort to kind of somehow lure them into a room and made them look at this, I could actually create a great discussion, and I could do something good with it, it’s just that … I don’t have any more time to put into this. … My primary obligation is to [my student] and to make sure that she gets a good Ph.D. … I mean whatever I could do with them was kind of extra. (Professor, Science Communication)

Of course, despite being packaged as discrete entities, projects do not start from blank slates. Instead, they have ambiguous relationships with larger scientific agendas, being simultaneously discrete and sustained by connections to commitments that lie outside their defined boundaries. To be credible—and therefore fundable—preliminary research has already been conducted and at least some investigations must be locked in and underway. This phenomenon, which in part results from competitive project-centred funding, creates what Aicardi et al. (2018) call a ‘synchrony mirage’ that makes it difficult for social scientists to have any direct impact on the research trajectories contained within a competitively awarded project because intellectual and material commitments have been made before it begins. A hopeful researcher discusses the challenges this phenomenon creates for responsible innovation:

Interviewer: ‘You’re at a point where you’re now trying to feed [your findings] back in?

Interviewee: ‘We’re beginning to do that, yeah. … With [this project], that’s the most appropriate mechanism, I think, because they’re already so far into the science. … The next case, hopefully, we’ll choose something where they’re more naïve in terms of their intellectual and engineering problems. (Researcher, STS)

This ‘discrete but connected’ nature of projects poses a challenge to the self-description of project-level responsible innovation as a process in which a scientific project is actively modulated to be more environmentally, politically, or socially aware than it would otherwise be. Social scientists must make commitments to be participants in projects, but the research explicitly located within them is at best a partial target for interventions. Instead, several of our interviewees emphasized that institutional change occurs over extended periods of inter-project time, and inter-generational time, through extended collaborations and by acting in peripheral spaces such as management meetings, workshops, conferences, classrooms, and chains of WhatsApp messages:

For me, a lot of it was very informal. It was part of that whole ethnographic goal of becoming part of the community, hanging out with people, having these corridor conversations. (Senior researcher, Anthropology)

And in the following statement a senior social scientist describes her success as cultivating ‘trusting relationships’ on which others could build:

Hopefully I can … pave the way for the next generation. … I spend a lot of energy in kind of making collaborations, and working with the political level. … So I’m on this sort of ladder of building trusting relationships. But hopefully other people can use those steps along the way to do things. (Professor, Science Communication)

Her, and others’, perceived successes came not from within the boundaries of projects, but from the interactions played out in sites beyond the project: management boards, review committees and conference panels. Or, as another senior researcher described, success, evaluated in terms of ‘being invited back,’ came from ‘putting myself where these people are going to be’ (Professor, Sociology). These infrastructural, interstitial spaces of science are widely acknowledged but partitioned out of the scientific project as sites of research and intervention, challenging the ability to form collectives across projects and especially across locales. Instead, focusing on the laboratory and its outputs becomes a cost of doing social scientific research under the auspices of responsible innovation, and one further imposition on an increasingly crowded ‘epistemic living space’ of academia (Felt, 2017).

## Institutional inventions: Governing beyond the project

As funders have given meaning to responsible innovation, they have propagated one particular mode of governing in which the people to be made responsible for the trajectories of science, technology and innovation are researchers, the best place for this work to be done is in their laboratories, research centres or universities, and the way in which it should be achieved is by mandating attached activities to natural or physical science projects as a condition of competitively awarded funding. This means that responsible innovation is both part of and subject to a set of project-centred dynamics. Through *framing*, the scientific endeavour is positioned as one of technological innovation to address pre-given societal needs, operating within a cycle of investment and promise (Joly et al., 2010, p. 24), and limiting the kinds of questions that can be asked of innovation. Through *valuing*, initially equivocal understandings of academic worth become concrete, making certain approaches to responsible innovation—and the labour associated with them—valuable. And through *partitioning*, academic work is divided, with responsibility assigned to some temporal and organizational arenas but not others. Collectively, these processes circumscribe the form that responsible innovation takes within contemporary research policy.

If a form of political life organized around technology-driven economic growth, liberal individualism, and rational management is characteristic of the late 20th and early 21st century, then responsible innovation might be viewed as one site in which its tendencies are made explicit. In responsible innovation, ideas of innovation governance are fused with dominant economic regimes of technology-driven growth, while understandings of innovation grounded in heterodox economic regimes or as something-other-than-technological are framed-out (de Saille & Medvecky, 2016; Lave et al., 2010). Pluralistic, collective, and situated approaches to engaging with questions of social and ecological responsibility are devalued and discouraged, particularly if they are seen to conflict with dominant models of value creation and extraction that will advance an individual career. The work that goes into fostering and building the relationships behind creative endeavours is partitioned as distinct from the work happening in efficient, well-managed project time and which will produce impactful outputs. As our interviewees show, those working under the label of responsible innovation are often viscerally aware of how they are both subjects and producers of the political logics encoded in mainstream investments into emerging science, technology, and innovation, and which are embodied in, enacted through, and sustained by responsible innovation as currently practised. In Felt’s (2017, p. 51) terms, responsible innovation is a kind of ‘moral glue’ that holds orthogonal promises of economic, societal, and scientific benefits together to allow broader imaginaries of innovative societies to unfold.

While not arbitrary, this arrangement of innovation governance is only one possibility amongst many. To explore some alternatives, we need new thinking that shifts the focus of responsible innovation to new people, sites, and modes of governing. We must consider the conditions through which this mode of governing research and innovation becomes possible, which is in part in the ways that administrators and managers in funding organizations think, design, and deploy policy instruments to govern the practices of those innovating. In other words, we can resist the acts of delegation to follow responsible innovation upstream into the carpeted corridors of science administration, to try and reformulate the technologies of government and the rationalities that they engender.

Our suggestion is to develop institutional inventions ‘in the wild’ (Callon et al., 2009, p. 69) that deliberately target policy instruments, patterns of thought, and social practices that shape the form of political life enshrined in, and reproduced through, contemporary innovation governance. Such inventions would move beyond long-articulated notions of institutional reflexivity, which call on those in power to understand how organizational commitments and arrangements create science and politics (Wynne, 2006), to recognize that the critical social sciences are already implicated in these processes, and actively attempt to design alternative arrangements (Smith et al., 2021). They resonate with Macnaghten’s (2020, p. 50) appeal for a ‘metis-informed social praxis’ which would lead ‘to the cultivation of parallel skills and capacities’ to understand ‘the social and cultural dynamics that shape and reinforce dominant understandings and framings’ in and around science, technology and innovation. And they would be driven by a broad set of questions: How could different aspects of the research and development enterprise be arranged to ensure that science and technology develop in ways that are more beneficial for people, animals, and the environment than they have been historically? Under what conditions should decision-making power over the trajectory of science and technology be devolved to relatively small groups, and when should it be opened to broad groups of citizens, stakeholders, and experts? How can social learning be normalized within the production of science, technology, and policy? These questions are core to the challenge of innovation governance and can only be addressed by considering the arrangement of actors, practices, and modes of reasoning within an institutional configuration.

We can outline four inflection points around which institutional inventions might be oriented—*agendas, calls, spaces*, and *evaluations*. Each of these four points represents an arena for dedicated policy experimentation and to varying degrees, each also contains established governance tools—strategic visions and delivery plans, funding calls, portfolio analyses, guidelines, tenders, proposal templates, evaluation panels, workshops, webinars, or reporting requirements, to name a few. Some immediate goals of a shift towards institutional invention would be to unsettle the dynamics colouring project-driven research and innovation by creating diverse frames for innovation to exist within, enabling new ways of valuing work to emerge and fostering connections between partitioned forms of academic life. However our four points map loosely rather than uniformly onto the project-based dynamics we have described above; taken together, experimentation and engagement with these targets would allow actors involved in the co-production of science and politics to voice how innovation might be made more responsive to societal needs, and to better understand the connections between democratic norms and scientific trajectories. In what follows, our goal is not to be exhaustive or tightly prescriptive; it is to draw existing but overlooked initiatives, sometimes conducted by our own informants, into the boundaries of responsible innovation to sketch general avenues for future research.

### Agendas: Diversifying framings

From an innovation governance perspective, the challenge for administrators is to generate either a diversity of different framings or to generate frames able to accommodate a range of different solutions (Stirling, 2014). Science administrators must develop new and strengthen existing mechanisms to actively pluralize decision-making about public research trajectories (Wynne, 2006). Understood in these terms, a goal of policy experimentation should be to expose existing commitments within research agendas and interrogate their assumptions: Why is one trajectory pursued over another? Who decided? And in response to which representations of a problem? It then becomes easier to open up, and potentially invest in, a broader plurality of trajectories (Stirling, 2016).

In research funding policy, several experiments to diversify the perspectives informing research programmes are instructive. These include direct experiments with innovation prizes, crowd-funding and relatively long-standing institutional structures, as in the Netherlands to allow citizens to not just be the consumers of research, but also to frame scientific questions (Leydesdorff & Ward, 2005). In 2018, Nesta, a British innovation think-tank, completed experiments in ‘inclusive innovation policy’ (Nesta, 2018). These relatively small-scale experiments in opening up agenda-setting processes can also be complemented with relatively large-scale attempts to mobilize members of the public to inform research agendas in nanotechnology and synthetic biology by the UK research councils (Delpy, 2011; Jones, 2008). In different ways and to varying degrees, these experiments unsettle the dominant frames of science policy: Prizes allocate money to prior rather than promised work, and participatory agenda-setting processes expose potential future trajectories to broader citizen scrutiny than would normally occur.

Similar goals can sometimes be achieved without direct citizen involvement. Such work can take the form of ‘uninvited public engagement’ and be mediated through existing agenda-setting processes, such as external review committees and open funding calls (Doubleday & Wynne, 2011). One contemporary example is the OpenPlant Research Centre, part of the UK Research Council’s Synthetic Biology for Growth Programme. OpenPlant prioritizes technological trajectories that do not rely on restrictive intellectual property arrangements, and in doing so has developed institutional innovations in the form of legal instruments, such as the Open Material Transfer Agreement (Kahl et al., 2018). These alternative commitments are explicitly framed in response to public concerns about the increasing privatization of plant science. Technical expertise and institutional reflexivity thus act to operationalize commonly articulated public concerns, such as about ownership and the distribution of benefits.

Finally, established but narrowly focused methodologies for making decisions about research funding priorities within administrative organizations can be repurposed to incorporate a broader set of concerns. For example, portfolio analysis, a common but usually econometric methodology, has recently been reformatted to map the relationship between technological trajectories and social need in health and food security domains (Cassi et al., 2017; Ciarli & Ràfols, 2019; Ràfols & Yegros, 2018). While technocratic rather than participatory, these proof-of-principle experiments demonstrate that by integrating new data with heterodox analytic goals, entrenched and credible policy practices can be reframed to expose existing political commitments alongside alternative options. Less clear is how to translate these new ways of visualising data into actionable funding decisions, a question likely only to be answered through concerted policy experimentation.

### Calls: Valuing interdisciplinarity differently

Many of the ambivalences with interdisciplinary and engaged research are the result of projects that over-prioritize instrumental logics such as accountability or innovation, whereby outsiders such as social scientists or stakeholders ensure the responsible behaviour of the natural sciences or accelerate the pace of product development (Barry et al., 2008). New modes of interdisciplinarity are emerging with alternative goals in mind. Operating under rubrics of ‘experimental collaboration’ (Calvert, 2013; Fitzgerald & Callard, 2015), ‘critical friendship’ (N. Rose, 2013) or ‘being alongside’ (Latimer, 2019), these new forms of interdisciplinarity aim to move from instrumental logics in which one discipline can be adorned with another and instead pursue more substantive relationships across disciplines.

Here, the targets of interdisciplinarity include the production of new knowledge that blends social and natural scientific studies of the same phenomena, the production of situated forms of reflexivity and even new technologies. As one senior scientist described, prioritizing responsible innovation for an extended period has led them to connect to an emerging network of interdisciplinary research within their university: ‘there’re [now] people coming out that we’re able to collaborate with’ (Professor, Synthetic Biology). This scientist, and others within the same organization, described their centre’s commitment to responsible innovation as ‘planting a red flag’ that people will be drawn to (Researcher, Synthetic Biology) and around which more substantive collaborations can be developed (see Guston, 2007).

An institutional challenge for funders in this regard is to not just mandate socio-technical integration, but also develop valuation structures that foster these more substantive forms of interdisciplinarity and that cut across projects. One obvious value structure is the evaluation of funding applications. A clear example comes not from a funding programme but an annual synthetic biology competition, iGEM, in which teams compete to use genetic engineering to develop a life sciences research project. The competition locks entrants into a narrow framing of problem and solution—something ‘out there’ in the world that synthetic biology technology can fix—but its organizers have fostered and supported ‘Human Practices’, their term for project-level responsible innovation, as a valuable approach by integrating it into the reward structure of the competition. A team cannot achieve a gold medal award, the highest level of recognition, without showing effective and substantive work in Human Practices (Balmer & Bulpin, 2013). In addition to formal competition rules, an ‘online hub’ is provided with resources and past examples deemed to be exemplary, over time generating new scientific norms.

Similar approaches have been adopted by European funding programmes such as ERA CoBioTech (Smith et al., 2019) and the Norwegian Research Council (Egeland et al., 2019). When assessing potential applications, ERA CoBioTech practiced ‘researcher equivalence’ between the natural and social sciences, and altered its administrative practices accordingly (Smith, Kamwendo, et al., 2021). Research partly funded by the Norwegian Research Council has drawn on learning from transdisciplinary research to collectively develop a rubric that captures the ‘quality’ of responsible innovation in the context of nanoremediation (Wickson & Carew, 2014). These policy experiments, developed between social scientists and science administrators, demonstrate that it is possible to develop and embed valuation structures that support non-instrumental modes of interdisciplinary research within the administrative fabric of funding programmes.

### Spaces: Making time for collective experimentation

Throughout the history of science and engineering, spaces for discussion have played a fundamental role in fostering social learning, institutional reflexivity, and new enactments of collective responsibility. Forums played vital roles in the creation (and domestication) of consensus-based decision-making structures to generate collective responsibility amongst French engineers in the 18th century (Graber, 2007). The Pugwash series of conferences are tightly interwoven with radical science movements and the Scientists for Social Responsibility organization that developed in the 1960s and ’70s (H. Rose & Rose, 1976). An analysis of the development of the UK Engineering and Physical Sciences Research Council’s (EPSRC) policy in responsible innovation emphasizes the importance of connected-but-temporary spaces (advisory committees, funding workshops and blogs) in allowing actors to develop shared registers (Murphy et al., 2016).

Of course, this final example illustrates that research funding organizations already go some way toward creating such spaces through agenda setting forums, training workshops and status update seminars (Kearnes, 2013). However, organizations that attempt to foster innovation governance have either neglected these spaces as an explicit target or, where explicit attempts have been made to convene such spaces, have tended to reinforce rather than interrogate existing institutional structures. Existing evaluative regimes, a narrow range of participants, or dominant logics of innovation, expertise, and interdisciplinary exchange have thwarted the opportunities that such discussion spaces offer (Murphy et al., 2016). As such, there remain relatively few examples of attempts by funding organizations to create spaces that allow individuals to move between the partitioned sites of research projects.

Nevertheless, three dimensions are crucial for building effective spaces for enacting collective responsibility. First, it must be possible to reflect on and challenge institutionalized commitments. Second, they must construct a temporal break for participants to pause and escape the ‘tyranny of urgency’ that colours contemporary scientific life (Joly et al., 2010). Third, they must be structured as ‘parallel spaces’ that allow participants to move in and out over time, and that are established not as unrestrained spaces of creativity but as ones imbued with a level of power through connections to governance and funding structures (Krzywoszynska et al., 2018). Conceptually, a rich body of research provides a foundation to build from. Trading zones (Gorman, 2010), hybrid forums (Callon et al., 2009) and competency groups (Landström et al., 2011) offer adequate foundations from which to develop spaces to experiment with new forms of responsibility in research policy contexts. Operationalized, each mobilizes an ethos of collective experimentation (Joly et al., 2010; Stilgoe, 2016) in which diverse participants assemble with relevant but not necessarily commensurable expertise to test and develop ideas with others in a structured setting.

### Evaluation: From accounting to learning

Our final focal point is the evaluation of science, technology, and innovation programmes. A key shift in the appraisal of evaluation has occurred in the past decade. Indicators, metrics and even qualitative forms of reporting are now recognized as constructed devices, designed to generate knowledge about science, technology, and innovation programmes (e.g. Barré, 2010; Müller & de Rijcke, 2017; Strathern, 2000). As with technologies in other settings, these constructivist perspectives recognize evaluation methodologies as producing particular forms of knowledge over others and being intrinsically interwoven to governing in the mode of ‘advanced liberal democracy’ (Miller & Rose, 1990). Both through design and accident, they embed value judgements and choices about which, or whose, knowledge is generated and conversely which, or whose, is not.

Recognising that evaluative methodologies and indicators are both designed and performative has created something of a florid air in research and innovation governance. One part of the mix consists of high-profile and widely lauded publications such as The San Francisco Declaration on Research Assessment (DORA), The Leiden Manifesto (Hicks et al., 2015) and The Metric Tide (Wilsdon et al., 2015), each with STS-informed recommendations for reform. Similarly, senior practitioners in the field such as Rémi Barré (2019) have made calls that those developing indicators have a ‘collective responsibility’ to ensure they are used to put science in the service of democracy. However, the performative strength of indicators and evaluation stems from being fully entrenched within the infrastructure of funding programmes. Thus, by far the major part of the mix is an ever-growing list of indicators and ways of using them. As a result, even when thoughtful, commissioned reports on indicators are developed, concluding, for instance, that they ‘cannot offer a general prioritized list of indicators’ and that instead diverse groups of actors should ‘devise their own processes of deliberation’ to generate context-specific indicators, the result still resembles a de-contextualized list from which administrators can, and do, pick (European Commission, 2015, p. 41).

To reiterate the earlier point, evaluation and indicators are parts of larger cultures of audit and accountability, which are themselves calculative activities inherent to many contemporary forms of governing (Miller & Rose, 1990; Porter, 1995; Strathern, 2000). Qualitative evidence and alternative framings of innovation, which for instance go beyond productivity and efficiency, are largely excluded from these calculations (Ràfols, 2019). There is some evidence that when employed in research policy, they play little role in developing social learning in the vein of administrative groups ‘puzzling together’ (Amanatidou et al., 2014). The pragmatic challenge for science administration, then, is to turn evaluation around and allow for plurality, experimentation, new modes of learning and, ultimately, intervening.

Quantitative methodologies can perform value and ensure accountability, but more recent integrative methodologies have begun to generate ways to evidence, reflect and learn from evaluation. Such approaches provide ways for research funding organizations to monitor their programmes differently and to rethink evaluation as an opportunity not just for compliance and accountability but also for learning and adaptation (Ràfols, 2019). This means tailoring indicators to their context of use and diversifying the kinds of information they capture to include qualitative information (see Felt et al., 2013) as well as alternative forms of quantitative information, such as the relative diversity of a field (Bozeman & Rogers, 2002). Recent developments in diversity mapping (Bone et al., 2020) and programme evaluations centred on human capabilities (O’Donovan et al., 2022) go some way to achieving this.

## Conclusion

With responsible innovation as an illustrative example, we have considered the limitations of dominant configurations of innovation governance as a way of governing science, technology, and innovation more responsibly than has historically been the norm. Collectively, the uptake of ideas such as responsible innovation may signify a desire to rebalance the focus of governance from the outputs of science to the cultures that produce science, technology, and innovation. Yet this will only come about if new social practices, patterns of thought and policy instruments are devised to reconfigure the institutions that shape those cultures.

In incorporating responsible innovation into their institutional vocabularies and logics, funding organizations have propagated one configuration amongst a range of possibilities. They have tended to ‘govern at a distance’ (Miller & Rose, 1990), indexing the idea with and through the tools that they have at their disposal—funding protocols, evaluation panels, established models of interdisciplinarity and accounting exercises. Because these tools were taken for granted, many of the aspirations of those working under the label of responsible innovation are circumscribed by processes that come with the tools of project-based governing: processes such as the framing, partitioning, and valuing that we have pointed to. In this configuration, responsible innovation is stabilizing, rather than opening up and potentially unsettling, established narratives and arrangements between science, politics, and society.

If governing through projects remains the dominant mode of enacting responsible innovation, the more transformational aspirations of innovation governance will continue to be undermined. The idea of institutional invention offers a heuristic to shift the emphasis of innovation governance away from researchers in projects and onto the existing tools and practices of governing used by science administrators. The four targets we have sketched out are starting points to begin to try disrupting habitual patterns of thought, discourse and action within science administration. They aim to create forms of governing that are sensitive to the kinds of social and political order they are creating and to the alternatives they are excluding (Stirling, 2016). They aim to create ruptures in normally scarce and staid environments in which things could be otherwise, if only for a moment. While each is modest, taking each with the other would begin to mark an embrace of responsibility on the part of research funding organizations, rather than the more common delegation that has coloured research and innovation policy to date.

The idea of institutional invention requires conceiving of science administrators, policy makers, and the organizations they comprise as actors with the agency, and inclination, to govern science and technology differently. It also continues a trend of bringing the critical social sciences into proximity with agents of power (Hackett & Rhoten, 2011). These two features raise many uncomfortable questions. For one, many studies of these organizations emphasize their inventiveness—in different ways, staff are already tuned to various social and political currents in the work that they do and are adept at assembling policies and procedures in ways that navigate these different pressures. We might point to the EPSRC’s reframing of the UK government’s impact agenda to incorporate the social as well as the economic (Kearnes & Wienroth, 2011). We might turn to managers in the Dutch National Program of Elderly Care and their careful experimentation to staging research in ways that presented a holistic rather than fragmented agenda (Oldenhof et al., 2022; Wehrens et al., 2022). Or we might point to the ways in which the European Research Area framework has been used by politicians and bureaucrats to further a vision of harmonization and integration, in obvious tension with ideals of pluralism (Lepori et al., 2013; Mahfoud, 2021; Pfister, 2016). The question then, is not whether staff in these organizations are responsive and inventive, but to what ends and whether those align with the political ends of STS scholars.

The histories of innovation governance themselves highlight how moments of institutional reflexivity, learning and change can emerge unexpectedly from combinations of external political pressure, organizational leaders and critical social scientists able to talk in many different registers (e.g. Doubleday & Wynne, 2011; Owen et al., 2021; Pallett & Chilvers, 2013; Smith et al., 2021b, Hartley, et al., 2021; Wynne, 2007). But they also show how policy windows close, how high levels of staff turnover make change difficult, that funders themselves are affected by the modes of governing deployed by their political masters, and that external events can just as easily create inertia—dynamics that work to produce situations in which the room for manoeuvre will be extremely narrow. These studies highlight how important extended and open-ended engagements between critical social sciences and practitioners are to capitalising on situations to reconfigure institutional landscapes, while cautioning against assuming that any singularly transformative outcome might be possible. While this might be read as deflationary, instead we understand it as a commitment to the complex, symmetrical and multidimensional analyses that characterize the best of STS. It is part of a commitment to collaborators labouring together to generate alternative, non-utopic futures with science and technology through policy, while also generating analyses of the work that goes into producing those futures (Masco, 2021). And it is only through such acts that the more substantive aspirations of innovation governance can be achieved.

## Notes

1. A lot has been written about the distinctions between responsible innovation and related terms like responsible research and innovation (RRI). As some have demonstrated, the distinction may matter in that policy makers have developed frameworks demarcating specific approaches to governance, and at times have prioritized distinct phrases (see Owen & Pansera, 2019). However, there is a lot of fluidity and slippage between the two terms, both in academic writing and practice. Here, our focus is on responsible innovation as illustrative of a broader idiom in innovation governance that circulates with a range of distinct but commensurate terms. We therefore refer primarily to responsible innovation and disambiguate RRI only when referring to the distinctive policy framework created by the European Commission.

2. Similar project-driven framings of responsible innovation can also be found in the European Commission’s Future and Emerging Technologies 2018-2020 work programme (European Commission, 2017b), the U.K.’s Synthetic Biology Research Centres (SBRCs) (BBSRC, 2013), the EPSRC’s Centres for Doctoral Training (EPSRC, 2018) as well as any number of multilateral European funding programmes (e.g. ERA CoBioTech, 2017; EuroNanoMed, 2020).
